# A pathological perspective to painful inguinal hernia: Report of two cases

**DOI:** 10.1016/j.ijscr.2021.106389

**Published:** 2021-09-06

**Authors:** Masato Narita, Koki Moriyoshi, Kentaro Goto, Ryoya Yamaoka, Takashi Yamaguchi

**Affiliations:** a Department of Surgery, National Hospital Organization, Kyoto Medical Center, 1-1 Fukakusamukaihata-cho, Fushimi-ku, Kyoto 612-8555, Japan; b Diagnostic Pathology, National Hospital Organization, Kyoto Medical Center, 1-1 Fukakusamukaihata-cho, Fushimi-ku, Kyoto 612-8555, Japan

**Keywords:** Chronic postoperative inguinal pain, Neuropathy, Laparoscopic hernia repair, Neuralgia

## Abstract

**Introduction:**

Preoperative inguinal pain (painful inguinal hernia) is a well-known factor associated with chronic postoperative inguinal pain (CPIP). However, it remains unclear what preventive measures should be taken in such patients.

**Case presentation:**

We report two patients with painful inguinal hernia who underwent pragmatic ilioinguinal nerve neurectomy during open anterior repair. The nerve was compressed by bulky spermatic cord lipoma in case 1 and by the hernia sac presenting over a few decades in case 2. Hematoxylin and eosin staining of the resected nerves revealed mucoid degeneration. Toluidine blue staining of resin-embedded nerve sections demonstrated that fully-myelinated axons had significantly decreased in case 1 and almost disappeared in case 2, indicating the development of massive demyelination of the ilioinguinal nerve in both cases.

**Discussion:**

In cases where the injured nerve is left in situ, CPIP may occur since demyelinating neuropathy sometimes becomes irreversible.

**Conclusion:**

Planned nerve resection via open anterior inguinal hernia repair may be an option to prevent CPIP in patients with painful inguinal hernia.

## Highlights

• Painful inguinal hernia is a known risk factor for CPIP; however, preventive measures remains unclear.
• Ilioinguinal nerves were massively demyelinated in two patients with painful inguinal hernia.
• Severe neuropathy induced by chronic compression of the ilioinguinal nerve may be associated with painful inguinal hernia.
• Planned nerve resection via anterior hernia repair is an option to prevent CPIP in patients with painful inguinal hernia.

## 1 Introduction

Chronic postoperative inguinal pain (CPIP) is the worst complication following inguinal hernia repair and significantly impairs patients' quality of life. Although laparoscopic hernia repair is reported to have a lower incidence of CPIP compared to open hernia repair, its frequency is still high. For example, data from the Herniamed Registry indicated that 9.5% of 20,004 patients reported pain on exercise at 1 year after transabdominal preperitoneal repair [1]. There are various risk factors involved in the development of CPIP, including the presence of inguinal pain before surgery [2]. However, it remains unclear what preventive measures should be taken in such patients [3]. In this case report, we describe the pathological specimens of pragmatically-resected ilioinguinal nerves during open anterior inguinal hernia repair in two patients with painful inguinal hernia. The aim of this report is to discuss the potential mechanisms of CPIP development and the potentially optimal surgical technique for such patients from a pathological perspective. This case report is reported in line with the SCARE criteria [4].

## 2 Case presentation

**Fig. 1:**
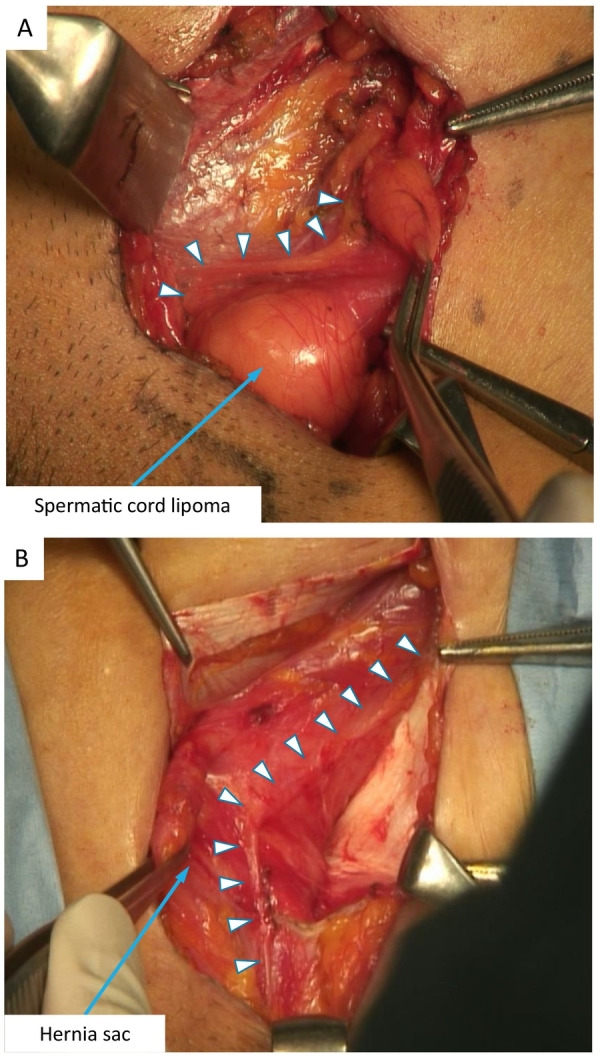
Images of the surgical procedures in *case 1* (A) and *case 2* (B). Arrowheads indicate the ilioinguinal nerves compressed by the bulky spermatic cord (A) and the hernia sac present over a few decades (B).

*Case 1* was a 35-year-old male with a body mass index of 28.3 kg/m^2^ who was referred to our hospital for treatment for a painful inguinal hernia. The patient described a baseline daily pain intensity of 0 on a 11-point numerical rating scale (NRS; 0/10), but experienced a sharp, shooting pain a few times per day with a maximum pain intensity of 5/10 while walking and performing sit-up exercises. The patient chose nonmesh repair. During surgery, the enlarged ilioinguinal nerve was identified just beside the spermatic cord lipoma (Fig. 1A). The nerve was resected as much as possible because it interfered with repair. The iliohypogastric nerve was identified and preserved, and Shouldice repair was performed for the indirect (L2) hernia. The patient's postoperative course was uneventful, and he was discharged from the hospital on the second postoperative day. The sharp, shooting pain completely disappeared immediately after surgery.

*Case* 2 was a 70-year-old female with painful left inguinal hernia that had been asymptomatic for 35 years. Her baseline daily pain intensity before surgery was 0/10, and she also experienced sharp, shooting pain a few times per day with a maximum pain intensity of 4/10 during walking. She also chose nonmesh repair. During surgery, an enlarged ilioinguinal nerve was observed and found to be compressed by the hernia sac (Fig. 1B). Because it interfered with repair, pragmatic neurectomy was performed. Shouldice repair was then performed for indirect (L3) hernia. Similar to *case 1*, the patient reported immediate relief from her preoperative pain after surgery. The postoperative course was uneventful, and she was discharged from the hospital on the second postoperative day.

**Fig. 2:**
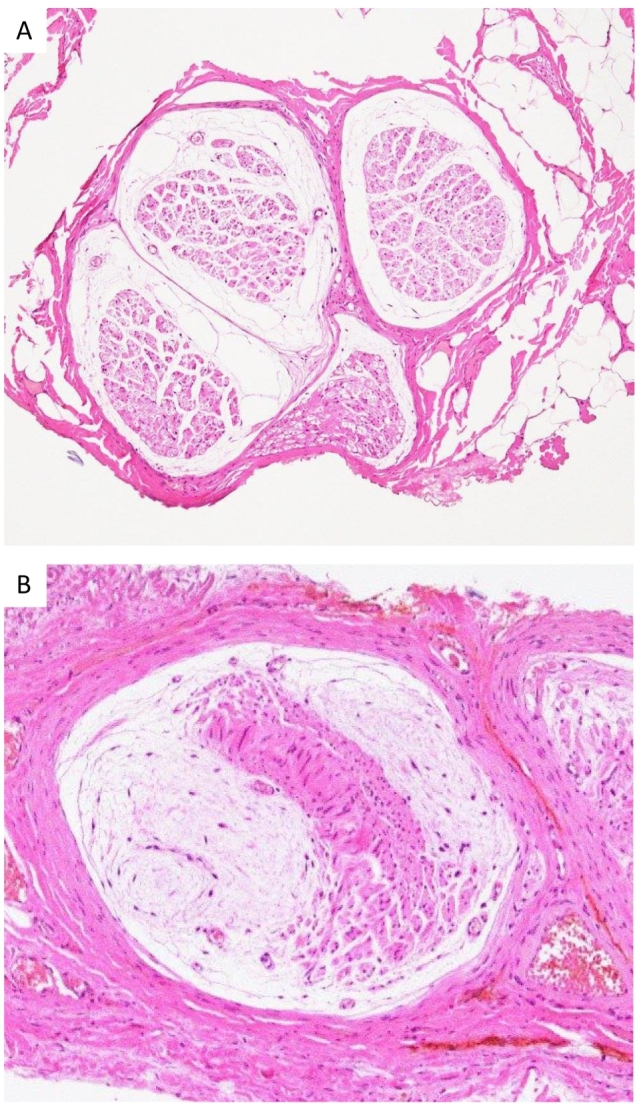
Hematoxylin and eosin staining of ilioinguinal nerve cross-sections in *case 1* (A) and *case 2* (B) Magnification at 100 × . Neural fibers markedly decreased and were displaced by the mucoid matrix, indicating mucoid degeneration in both cases.

**Fig. 3:**
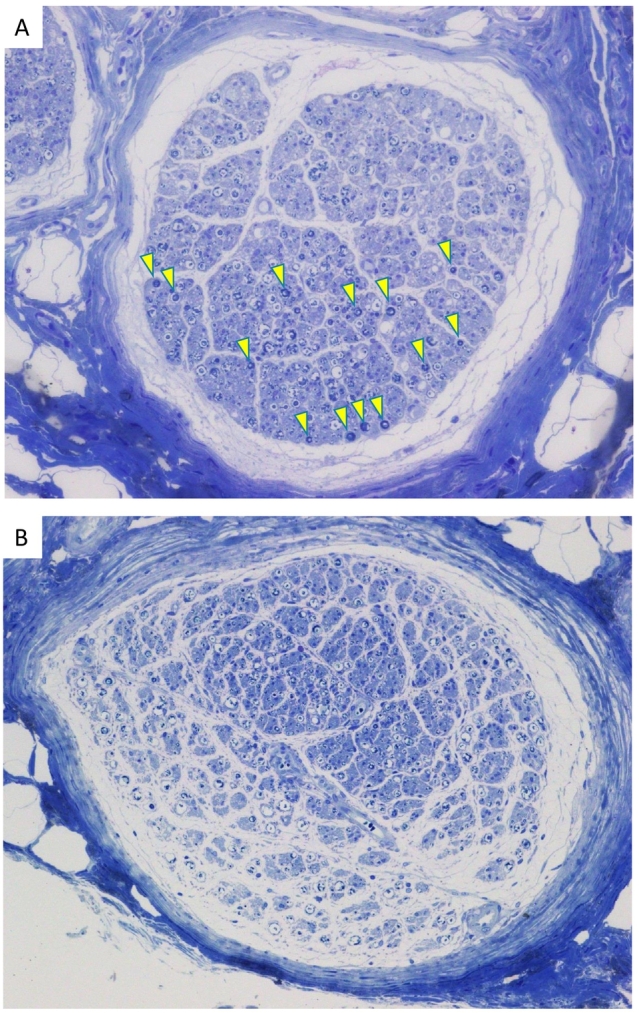
Toluidine blue staining of resin-embedded ilioinguinal nerve sections in *case 1* (A) and *case 2* (B). Magnification at 100 × . The myelin sheath was visualized as thick blue ring, as shown by arrowheads in *case 1 (*A). Fully-myelinated fibers were markedly decreased in case 1 (A) and almost lost in *case 2* (B). (For interpretation of the references to colour in this figure legend, the reader is referred to the web version of this article.)

Hematoxylin and eosin staining of the nerve cross-sections (magnification at 100×) demonstrated mucoid degeneration (Fig. 2A and B). Inflammation or atypical cells were not observed in nerve fibers or in the surrounding fat tissue. Toluidine blue staining of resin-embedded nerve sections, which is a reproducible method for qualitative and quantitative assessments of peripheral nerves, enabling visualization of the myelin sheath as thick blue ring [5] clearly demonstrated significant decreases in fully-myelinated axons in *case 1* and near disappearance in *case 2* (Fig. 3A and B), indicating the development of massive demyelination of the ilioinguinal nerve in both cases. In both cases, pain had completely disappeared (NRS: 0/10) 3 months post-surgery, and so periodical follow-up was discontinued.

## 3 Discussion

In the present case report, pathological findings revealed mucoid degeneration and demyelination of the ilioinguinal nerve in two cases. Mucoid degeneration can develop on all peripheral nerves, particularly those that must be preserved for motor function, e.g., the sciatic, upper extremity, and peroneal nerves [6], [7]. Mucoid degeneration usually appears as a response to chronic nerve injury, chronic compression, or repetitive microtrauma to the nerve [7]. Local pain, paresthesia, and nerve paralysis are common symptoms. Neuropathy of the ilioinguinal nerves was confirmed by toluidine blue staining of resin-embedded nerve sections in both cases, which revealed massive demyelination. These pathological findings suggest that the preoperative inguinal pain was neurogenic, which results from neuropathy of the ilioinguinal nerve. Considered along with the intraoperative findings, these results suggest that severe neuropathy was induced by chronic compression of ilioinguinal nerves due to a bulky spermatic cord lipoma in *case 1* and inguinal hernia presenting over a few decades in *case 2*. These findings are in line with previous studies reporting that compression of the ilioinguinal nerve by the external inguinal ring was involved in painful inguinal hernia [8], [9], [10].

It is expected that most injured nerves heal spontaneously after removing mechanical compression. However, some instances may become irreversible and lead to the development of CPIP. This may be the answer to the question of why the incidence of CPIP is still high following laparoscopic hernia repair. In such conditions, planned nerve resection via open anterior inguinal hernia repair may be an option to not only get rid of neuropathic pain but also to prevent persistent pain after repair.

How should we distinguish neuropathic pain from pain due to incarceration? Patients' descriptions of pain characteristics may be helpful for identification of this type of pain prior to hernia repair. In the present study, both cases described their pain as “sharp, shooting,” which is a specific description of neurogenic pain [11].

## 4 Conclusions

Planned nerve resection via open anterior inguinal hernia repair may be a suitable option for preventing CPIP in patients with painful inguinal hernia. However, the findings of this case report are not representative of the general population. Thus, a prospective study with large sample size is needed to address questions, such as how many patients with painful inguinal hernia are neurogenic, what patients have severe neuropathy, and what an optimal surgical technique would be for such patients.

## Abbreviations

CPIP: chronic postoperative inguinal pain
NRS: numerical rating scale

## Ethics approval and consent to participate

This article does not require ethical approval.

## Consent for publication

Written informed consent was obtained from the patient for publication of this case report and accompanying images. A copy of the written consent is available for review by the Editor-in-Chief of this journal on request.

## Availability of data and material

Not applicable.

## Funding

The author(s) received no financial support for the research, authorship, and/or publication of this article.

## Provenance and peer review

Not commissioned, externally peer-reviewed.

## Guarantor

Masato Narita

## CRediT authorship contribution statement

MN: Management of these cases and preparing the manuscript

KM: Pathological assessment and preparing the manuscript

KG: Management of these cases and review of the manuscript

RY: Management of these cases and review of the manuscript

RM: Management of these cases and review of the manuscript

HH: Critical appraisal and review of the manuscript

TY; Critical appraisal and review of the manuscript

## Declaration of competing interest

The author(s) declared no competing interest with respect to the research, authorship, and/or publication of this article.
